# Genomic Landscape and Clinical Features of Advanced Thyroid Carcinoma: A National Database Study in Japan

**DOI:** 10.1210/clinem/dgae271

**Published:** 2024-04-17

**Authors:** Soji Toda, Yukihiko Hiroshima, Hiroyuki Iwasaki, Katsuhiko Masudo

**Affiliations:** Department of Endocrine Surgery, Kanagawa Cancer Center, Kanagawa 241-8515, Japan; Department of Breast and Thyroid Surgery, Yokohama City University Medical Center, Kanagawa 232-0024, Japan; Department of Cancer Genome Medicine, Kanagawa Cancer Center, Kanagawa 241-8515, Japan; Research Institute Division of Advanced Cancer Therapeutics, Kanagawa Cancer Center Research Institute, Kanagawa 241-8515, Japan; Department of Endocrine Surgery, Kanagawa Cancer Center, Kanagawa 241-8515, Japan; Department of Endocrine Surgery, Kanagawa Cancer Center, Kanagawa 241-8515, Japan

**Keywords:** thyroid carcinoma, gene, comprehensive genetic profiling test, mutations, fusions, targeted therapy

## Abstract

**Context:**

The relationship between the genomic profile and prognosis of advanced thyroid carcinoma requiring drug therapy has not been reported.

**Objective:**

To evaluate the treatment period and overall survival time for each genetic alteration in advanced thyroid carcinoma that requires drug therapy.

**Methods:**

We conducted a retrospective observational study using a national database in Japan, which included 552 cases of thyroid carcinoma out of 53 543 patients in the database.

**Results:**

The database included anaplastic thyroid carcinoma (23.6%), poorly differentiated thyroid carcinoma (10.0%), and differentiated thyroid carcinoma (66.4%). The most common genetic abnormalities were *TERT* promoter (66.3%), *BRAF* (56.7%), and *TP53* (32.2%). The typical driver genes were *BRAF* V600E (55.0%), *RAS* (18.5%), *RET* fusion (4.7%), *NTRK* fusion (1.6%), and *ALK* fusion (0.4%). The most common regimen was lenvatinib, and the time to treatment failure was not different despite the presence of *BRAF* or *RAS* mutations. In differentiated thyroid carcinoma and poorly differentiated thyroid carcinoma, *TP53* alterations independently predicted worse overall survival (hazard ratio = 2.205, 95% confidence interval: 1.135-4.283). In anaplastic thyroid carcinoma, no genetic alterations were associated with overall survival.

**Conclusion:**

Genetic abnormalities with treatment options were found in 62.7% of advanced thyroid carcinomas. *TP53* abnormality was an independent poor prognostic factor for overall survival in differentiated thyroid carcinoma. The time to treatment failure for lenvatinib was not different based on genetic profile.

Tumors originating from thyroid follicular cells are the most common type of thyroid carcinoma and are classified into BRAF-like malignancies, represented by papillary thyroid carcinomas (PTCs) with many morphological subtypes, and RAS-like malignancies, represented by invasive encapsulated follicular variant PTC and follicular thyroid carcinoma (1). In addition to *BRAF* and *RAS* mutations, *RET* and *NTRK* fusion genes are typical driver genes that are acquired early during PTC (2). However, *RAS* mutations are observed in 30% to 45% of follicular thyroid carcinoma (FTC) cases, and *PAX8/PPAR* fusion is the second most common genetic abnormality in approximately 30% of cases (3, 4). In addition to these early-acquired genetic abnormalities, accumulation of *TERT* promoter mutations and *TP53* abnormalities are thought to increase the malignancy of the tumor and result in anaplastic transformation (5).

Most thyroid carcinomas can be controlled with surgery and radioactive iodine therapy. However, in a small number of patients, the disease progresses despite these treatments, and drug therapy is required. Multikinase inhibitors, such as sorafenib and lenvatinib, have been used successfully to treat thyroid carcinoma (6, 7), and specific inhibitors for genetic abnormalities acquired early, such as *BRAF*, *RET*, and *NTRK* alterations, are effective (8-12). Thyroid carcinoma is characterized by many genetic abnormalities that can be therapeutic targets. However, large-scale studies of drug treatments and genetic profiles have not been reported.

A national database in Japan, the C-CAT, was established to register advanced cancer cases wherein comprehensive genetic profiling (CGP) tests were performed. The C-CAT includes clinical data sent by the participating hospitals and the sequence data of CGP tests. It also facilitates the appropriate secondary use of repository data for researchers in academic institutions and industries upon the consent of the patients (13, 14). Most thyroid carcinomas have a good prognosis, and genetic profile information in advanced cases is valuable. Therefore, we decided to use the C-CAT database to search for the relationship between prognosis and genetic abnormalities in advanced thyroid carcinomas that require drug therapy.

## Materials and Methods

### Study Population

This retrospective observational study collected clinical and genomic data from the C-CAT database (ver. 20230622) in July 2023. All data used for the analysis were obtained from patients who provided written informed consent and consent for future research use when registering for the database. In Japan, national health insurance coverage for the CGP tests was introduced in June 2019 for patients with solid cancers wherein no standard treatment is available or standard treatment is expected to be completed. In thyroid cancer patients, the main targets for CGP tests are differentiated thyroid carcinomas (DTCs) that cannot be controlled by surgery, radioactive iodine therapy, or multikinase inhibitors and unresectable anaplastic thyroid carcinoma (ATC). Therefore, patients registered in the database include only those with advanced cancer. At the time of data collection, 3 CGP tests had been approved; OncoGuide™ NCC Oncopanel System (NCC) (Sysmex Co., Ltd., Kobe, Japan), FoundationOne® CDx (F1CDx), and FoundationOne® Liquid CDx (F1L) (Foundation Medicine Inc., Cambridge, MA, USA). F1CDx and F1L are DNA-based next-generation sequencing panel tests that include 324 genes and use tumor tissues and blood, respectively. NCC is also a DNA-based next-generation sequencing panel test that includes 124 genes using tumor tissue and blood.

From June 2019 to June 2023, 586 out of 53 543 cases registered in the C-CAT database cases had thyroid malignancy. Medullary thyroid carcinoma, carcinoma showing thymus-like differentiation, intrathyroidal thymic carcinoma, mucoepidermoid carcinoma, mucinous carcinoma, or with unknown histology type were excluded to qualify malignant tumors derived from the follicular epithelium. The remaining 552 cases consisting of ATC, DTC, and poorly differentiated thyroid carcinoma (PDTC) were analyzed. This study was approved by the Kanagawa Cancer Center Institutional Review Board (no. 2022-144) and by the review board of C-CAT (C-CAT control no. CDU2023-005N).

### Methods

The following background characteristics were collected from the C-CAT database: pathological diagnosis, age, sex, smoking history, excessive alcohol use, Eastern Cooperative Oncology Group performance status (ECOG-PS), metastatic organs, pharmacotherapy history, and CGP testing methods. Tumor mutation burden (TMB) and microsatellite instability (MSI) status were estimated via the CGP tests. Cases with a TMB of level 10 or with high mutations per megabase were defined as TMB-high. Druggable gene alterations include *NTRK* fusions, *RET* fusions, *ALK* fusions, *BRAF* V600E mutation, TMB-high, or MSI-high.

To evaluate treatment efficacy, the overall response rate (ORR), disease control rate (DCR), and time to treatment failure (TTF) were estimated. ORR was defined as the proportion of the patients with a complete or partial response. DCR was defined as the proportion of the patients with a complete response, partial response, or stable disease. These were evaluated by the physicians according to the Response Evaluation Criteria In Solid Tumors version 1.1. TTF was defined as the time from the start of treatment to the end of treatment or death due to any cause. Overall survival (OS) was measured from the start of treatment to the date of death, regardless of cause.

The clinical annotations provided by C-CAT were used to assess genomic alterations (14). To analyze each alteration, we consulted the Evidence Database of C-CAT, which contains clinical evidence for specific mutations, encompassing categories such as predictive, prognostic, diagnostic, predisposing, pathogenic, and oncogenic information. The data are acquired from C-CAT's proprietary and from publicly accessible databases, including Clinical Interpretation of Variants in Cancer (15), BRCA Exchange (16), ClinVar (17), and Catalogue of Somatic Mutations in Cancer (18). Genes annotated as “oncogenic,” “pathogenic,” “likely oncogenic,” and “likely pathogenic” were extracted. For some patients, clinical data were missing, likely from encoding errors from each attending physician. Therefore, the analyses included patients with fixed data.

### Statistical Analysis

Fisher's exact test was used to compare all categorical variables. Survival analysis was performed with 95% confidence interval (CI) using the Kaplan–Meier method and compared using the log-rank test. A Cox proportional hazard regression model was used for multivariable analysis to estimate the association between genetic alterations and OS, using variables with *P* < .2 on univariable analysis. Statistical significance was set at *P* < .05. Statistical analyses were conducted using R version 4.3.1 (R Foundation for Statistical Computing, Vienna, Austria). The oncoplot was drawn using maftools (version 2.17.0) (19).

## Results

### Clinical Characteristics

**Figure 1.**
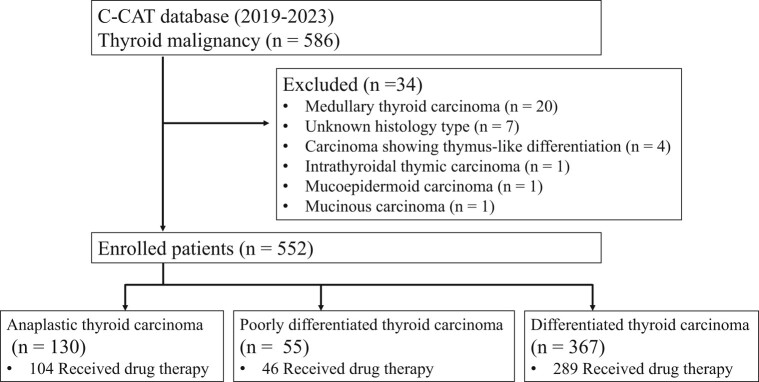
Flow diagram of the study.

Among 552 patients in this study, 130 (23.6%) had ATC, 307 (55.6%) had PTC, 55 (10.0%) had FTC, 5 (0.9%) had well-differentiated thyroid carcinoma, and 55 (10.0%) had PDTC. Drug therapy was received by 104 (80.0%) patients with ATC, 289 (78.7%) with DTC, and 46 (83.6%) with PDTC (Fig. 1). The clinical characteristics of all patients at the time of registration according to their pathological types are summarized in Table 1. The median age was 71 years (minimum: 4, first quartile: 61, third quartile: 75, maximum: 89), with 310 (56.2%) females 242 (43.8%) males. ECOG-PS was 0 or 1 in most cases. Metastasis was seen in 522 (94.5%) patients, with the most common metastatic sites being the lung in 381 patients (73%), lymph node in 306 patients (58.6%), and bone in 143 patients (27.4%).

**Table 1.** Patient characteristics

	ATC	PTC	FTC	WDTC	PDTC	Total	*P*-value*^a^*
	n = 130	n = 307	n = 55	n = 5	n = 55	n = 552	
Age (median)	72	70	67	61.5	72		
Sex, n (%)							.129
Male	49 (37.7)	142 (46.3)	25 (45.5)	2 (40)	24 (43.6)	242 (43.8)	
Female	81 (62.3)	165 (53.7)	30 (54.5)	3 (60)	31 (56.4)	310 (56.2)	
Smoking history, n (%)	52 (40)	114 (37.1)	15 (27.3)	1 (20)	25 (45.5)	207 (37.5)	.535
Excessive alcohol use, n (%)	20 (15.4)	35 (11.4)	4 (7.3)	0	6 (10.9)	65 (11.8)	.116
ECOG-PS, n (%)*^b^*							.748
0, 1	122 (93.8)	299 (97.4)	53 (96.4)	5 (100)	52 (94.5)	531 (96.2)	
2, 3	4 (3.1)	5 (1.6)	2 (3.6)	0	3 (5.5)	14 (2.5)	
Presence of metastasis, n (%)	116 (89.2)	293 (95.4)	54 (98.2)	5 (100)	54 (98.2)	522 (94.6)	.**006**
Metastatic organs, n (%)							
Lung	82 (70.7)	224 (76.5)	31 (57.4)	4 (80)	40 (74.1)	381 (73)	.104
Lymph node	67 (57.8)	188 (64.2)	18 (33.3)	2 (40)	31 (57.4)	306 (58.6)	.315
Bone	12 (10.3)	64 (21.8)	40 (74.1)	3 (60)	24 (44.4)	143 (27.4)	**<**.**001**
Liver	6 (5.2)	13 (4.4)	7 (13)	0	4 (7.4)	30 (5.7)	.825
Pleura	4 (3.4)	22 (7.5)	2 (3.7)	1 (20)	2 (3.7)	31 (5.9)	.192
Brain	1 (0.9)	19 (6.5)	2 (3.7)	1 (20)	4 (7.4)	27 (5.2)	.**009**
Number of regimens, n (%)							.**002**
0, 1	120 (92.3)	252 (82.1)	45 (81.8)	4 (80)	41 (74.6)	462 (83.7)	
>2	10 (7.7)	55 (17.9)	10 (18.2)	1 (20)	14 (25.4)	90 (16.3)	
Genomic profiling test, n (%)							.155
F1CDx	111 (85.4)	254 (82.7)	39 (70.9)	3 (60)	43 (78.2)	450 (81.5)	
F1L	7 (5.4)	28 (9.1)	11 (20)	2 (40)	6 (10.9)	54 (9.8)	
NCC	12 (9.2)	25 (8.1)	5 (9.1)	0	6 (10.9)	48 (8.7)	
Sample collection method, n (%)							.**017**
Surgery	86 (66.2)	230 (74.9)	31 (56.4)	1 (20)	40 (72.7)	388 (70.3)	
Biopsy	37 (28.5)	49 (16)	13 (23.6)	2 (40)	9 (16.4)	110 (19.9)	
Specific collection site, n (%)							**<**.**001**
Thyroid	94 (72.3)	88 (28.7)	18 (32.7)	0	26 (47.3)	226 (40.9)	
Lymph node	19 (14.6)	107 (34.9)	8 (14.5)	2 (40)	14 (25.5)	150 (27.2)	
Lung	3 (2.3)	27 (8.8)	5 (9.1)	0	3 (5.5)	38 (6.9)	
Soft tissue	3 (2.3)	20 (6.5)	1 (1.8)	0	3 (5.5)	27 (4.9)	
Bone	0 (0)	5 (1.6)	9 (16.4)	0	0	14 (2.5)	
Liver	2 (1.5)	5 (1.6)	2 (3.6)	0	1 (1.8)	10 (1.8)	
Other	2 (1.5)	27 (8.8)	1 (1.8)	1 (20)	2 (3.6)	33 (6)	

Abbreviations: ATC, anaplastic thyroid carcinoma; ECOG-PS, Eastern Cooperative Oncology Group performance status; F1CDx, FoundationOne® CDx; F1L, FoundationOne® Liquid CDx; FTC, follicular thyroid carcinoma; NCC OncoGuide™, NCC Oncopanel System; PDTC, poorly differentiated thyroid carcinoma; PTC, papillary thyroid carcinoma; WDTC, well-differentiated thyroid carcinoma.

*P* < .05 are shown in bold.

^*a*^ATC vs non-ATC.

^*b*^Data available for 126 patients with ATC and 304 patients with PTC.

When comparing ATC to DTC and PDTC, we noted no significant differences in terms of sex, smoking history, excessive alcohol use, or ECOG-PS. Metastasis, particularly to the bone and brain, was significantly less frequent in ATC. Patients with ATC also had fewer prior treatment regimens. In terms of CGP testing, F1CDx, F1L, and NCC tests were performed in 450 (81.5%), 54 (9.8%), and 48 (8.7%) cases, respectively. As a method of tissue collection, biopsies were performed most frequently in ATC, with the thyroid being the most common site for tissue collection. For PTC, the most common site of tissue collection was the lymph nodes.

### Genomic Characteristics

**Figure 2.**
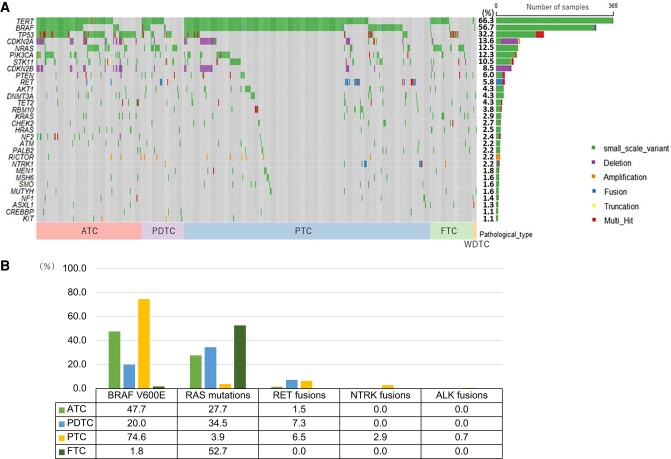
(A) Overview of the most common genomic alterations. The right bar shows the number and the frequency of cases with gene alterations in each gene. (B) The frequency of representative driver genes. Abbreviations: ATC, anaplastic thyroid carcinoma; FTC, follicular thyroid carcinoma; PDTC, poorly differentiated thyroid carcinoma; PTC, papillary thyroid carcinoma; WDTC, well-differentiated thyroid carcinoma.

The most common genetic abnormalities across all tissue types were *TERT* promoter (n = 336, 66.3%), *BRAF* (n = 313, 56.7%), and *TP53* (n = 178, 32.2%) (Fig. 2A). The typical driver genes were *BRAF* V600E mutation (n = 304, 55.1%), *RAS* mutation (n = 102, 18.5%), *RET* fusion (n = 26, 4.7%), *NTRK* fusion (n = 9, 1.6%), and *ALK* fusion (n = 2, 0.4%). Among patients with PTC, *BRAF* V600E mutation was the most common (in 229 cases, 74.6%), followed by *RET* fusion (in 20, 6.5%), *RAS* mutation (in 12, 3.9%), and *NTRK* fusion (in 9, 2.9%). Of the patients with FTC, 19 cases (34.5%) had *NRAS* mutations, 6 cases (10.9%) had *KRAS* mutations, and 4 cases (7.3%) had *HRAS* mutations. Because the gene panel tests in this study could not reveal *PPARγ/PAX8* fusion, other frequently occurring driver genes were not detected. Of the patients with PDTC, 19 cases (34.5%) had *RAS* mutations, 11 cases (20%) had *BRAF* V600E mutation, and 4 cases (7.3%) had *RET* fusion. Of the patients with ATC, 62 cases (47.7%) had *BRAF* V600E mutation, 36 cases (27.7%) had *RAS* mutations, and 2 cases (1.5%) had *RET* fusion, which suggests that some cases of PTC and FTC transformed into ATC as inheriting driver genes (Fig. 2B).

Genetic alterations that were significantly common in ATC included tumor suppression genes (eg, *TP53*, *NF2*, and *RB1*), PI3K/AKT pathway genes, cell cycle pathway genes, and epigenetic modification genes (eg, *TET2*) (Table 2). No genetic alterations had significantly higher frequency in PDTC and DTC, except for *RBM10,* which is an RNA-binding protein that regulates the alternative splicing of pre-mRNA, suggesting that gene mutations are accumulated more in ATC. Additionally, TMB was significantly higher in ATC than the others, suggesting genomic instability of ATC (mean: 3.02 vs 1.95, *P* < .001). TMB-high was seen in 12 cases (2.2%) of all thyroid carcinomas including 8 cases (6.2%) of ATC, 1 case (1.8%) of PDTC, and 3 cases (1.0%) of PTC. However, MSI-high cancer was not seen in any pathological type. The frequency of druggable gene alterations was 52.3% (68/130) in ATC, 29.1% (16/55) in PDTC, 84.7% (260/307) in PTC, and 1.8% (1/55) in FTC.

**Table 2.** Genes more frequently altered in ATC than in differentiated and PDTC

Gene	Prevalence, n (%)		*P*-value
	ATC	DTC and PDTC	
*TP53*	103 (79.2)	75 (17.8)	<.001
*CDKN2A*	34 (26.2)	41 (9.7)	<.001
*PIK3CA*	27 (20.8)	41 (9.7)	.002
*NRAS*	24 (18.5)	45 (10.7)	.023
*CDKN2B*	22 (16.9)	25 (5.9)	<.001
*NF2*	14 (10.8)	1 (0.2)	<.001
*TET2*	10 (7.7)	14 (3.3)	.046
*RB1*	5 (3.8)	1 (0.2)	.003
*TSC1*	4 (3.1)	2 (0.5)	.03
*KDR*	3 (2.3)	1 (0.2)	.042

Abbreviations: ATC, anaplastic thyroid carcinoma; DTC, differentiated thyroid carcinoma; PDTC, poorly differentiated thyroid carcinoma.

### Treatment

Among the registered treatment regimens, the most common treatment was lenvatinib, followed by sorafenib. In ATC, lenvatinib was the most common, followed by paclitaxel. Although details of the investigational drugs are unknown, there was little use of combined therapy with BRAF and MEK inhibitors, which were not approved in Japan (Table 3). We investigated the response rate for lenvatinib, which is the most common first-line regimen. The ORR was 17.4% (12/69) in ATC, 51.9% (84/162) in PTC, 41.0% (16/39) in FTC, and 50.0% (14/28) in PDTC. The DCR was 52.2% (36/69) in ATC, but this was 82.8% (192/232) in DTC and PDTC. ORR and DCR are significantly lower in ATC than in DTC and PDTC (*P* < .001, Table 4).

**Table 3.** Administered regimen

Number of patients who received drug therapy, n (%)	ATC	PTC	FTC	WDTC	PDTC	Total
	n = 104	n = 237	n = 48	n = 4	n = 46	n = 439
Lenvatinib	75 (72.1)	186 (78.5)	41 (85.4)	3 (75)	36 (78.3)	341 (77.7)
Sorafenib	1 (1)	59 (24.9)	11 (22.9)	1 (25)	14 (30.4)	86 (19.6)
Selpercatinib	0 (0)	2 (0.8)	0 (0)	0 (0)	1 (2.2)	3 (0.7)
Larotrectinib	0 (0)	3 (1.3)	0 (0)	0 (0)	0 (0)	3 (0.7)
Pembrolizumab	2 (1.9)	1 (0.4)	0 (0)	0 (0)	1 (2.2)	4 (0.9)
Nivolumab + lenvatinib	1 (1)	0 (0)	0 (0)	0 (0)	0 (0)	1 (0.2)
Dabrafenib + trametinib	3 (2.9)	1 (0.4)	0 (0)	0 (0)	0 (0)	4 (0.9)
Encorafenib + binimetinib	1 (1)	0 (0)	0 (0)	0 (0)	0 (0)	1 (0.2)
Paclitaxel	15 (14.4)	2 (0.8)	0 (0)	1 (25)	2 (4.3)	20 (4.6)
Other chemotherapy	4 (3.8)	2 (0.8)	0 (0)	0 (0)	1 (2.2)	7 (1.6)
Investigated agent	5 (4.8)	25 (10.5)	1 (2.1)	0 (0)	6 (13)	37 (8.4)

Abbreviations: ATC, anaplastic thyroid carcinoma; FTC, follicular thyroid carcinoma; PDTC, poorly differentiated thyroid carcinoma; PTC, papillary thyroid carcinoma; WDTC, well-differentiated thyroid carcinoma.

**Table 4.** Best overall response of first-line lenvatinib

Response, n (%)	ATC	PTC	FTC	PDTC	*P*-value*^a^*
	n = 69	n = 162	n = 39	n = 28	
Complete response	2 (2.9)	0 (0)	0 (0)	0 (0)	
Partial response	10 (14.5)	84 (51.9)	16 (41)	14 (50)	
Stable disease	24 (34.8)	51 (31.5)	16 (41)	8 (28.6)	
Progressive disease	8 (11.6)	7 (4.3)	1 (2.6)	1 (3.6)	
Not evaluable	25 (36.2)	20 (12.3)	6 (15.4)	5 (17.9)	
Overall response rate	12 (17.4)	84 (51.9)	16 (41.0)	14 (50.0)	**<**.**001**
Disease control rate	36 (52.2)	135 (83.3)	32 (82.1)	22 (78.6)	**<**.**001**

Abbreviations: ATC, anaplastic thyroid carcinoma; FTC, follicular thyroid carcinoma; PDTC, poorly differentiated thyroid carcinoma; PTC, papillary thyroid carcinoma.

^*a*^ATC vs non-ATC.

**Figure 3.**
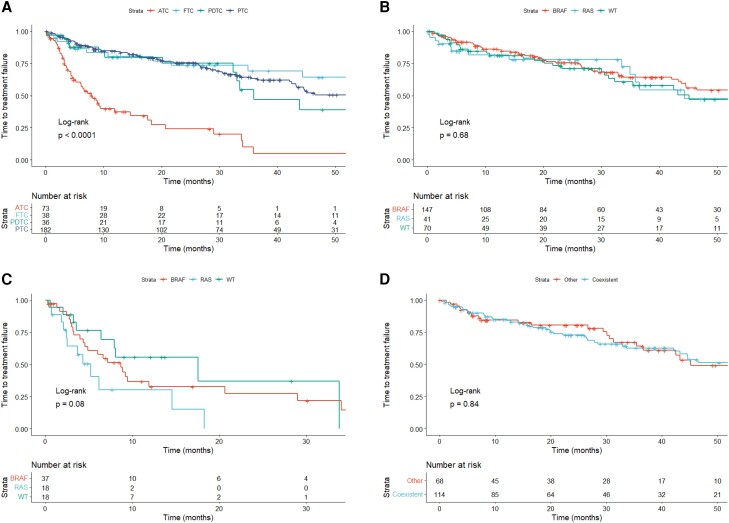
Kaplan–Meier curves of TTF according to first-line lenvatinib therapy. (A) TTF by histological types; (B) TTF of DTC and PDTC classified as *BRAF*-positive, *RAS*-positive, and both negative; (C) TTF of ATC classified as *BRAF*-positive, *RAS*-positive, and both negative; (D) TTF of PTC by *BRAF* and *TERT* promoter mutations. Abbreviations: ATC, anaplastic thyroid carcinoma; DTC, differentiated thyroid carcinoma; FTC, follicular thyroid carcinoma; PDTC, poorly differentiated thyroid carcinoma; PTC, papillary thyroid carcinoma; TTF, time to treatment failure; WT, wild-type.

Afterward, we examined TTF for first-line lenvatinib. The TTF was significantly shorter in cases with ATC than with DTC and PDTC (*P* < .0001, Fig. 3A). In DTC and PDTC patients, the median TTF was not significantly different among cases with specific genetic mutations (*P* = .68, Fig. 3B). The median TTF across ATC cases with genetic mutations also had no significant differences (*P* = .08, Fig. 3C). In PTC, TTF for first-line lenvatinib had no significant differences according to concurrent of *BRAF* and *TERT* promotor mutations (*P* = .84, Fig. 3D).

### OS

**Figure 4.**
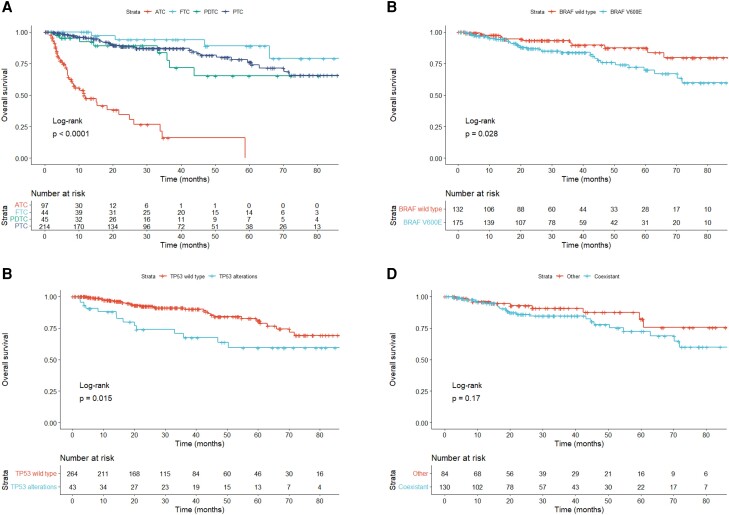
Kaplan–Meier curves of OS. (A) OS by histological types; (B) OS of DTC and PDTC by *BRAF* V600E mutation status; (C) OS of DTC and PDTC by *TP53* abnormality; (D) OS of PTC by *BRAF* and *TERT* promoter mutations. Abbreviations: ATC, anaplastic thyroid carcinoma; DTC, differentiated thyroid carcinoma; FTC, follicular thyroid carcinoma; OS, overall survival; PDTC, poorly differentiated thyroid carcinoma; PTC, papillary thyroid carcinoma.

The Kaplan–Meier curves of OS for each pathological type are shown in Fig. 4A. OS was significantly shorter in ATC, with a median OS of 11.5 months (95% CI 7.9-21.7), compared to FTC [not accessible (NA), 95% CI 66.0–NA], PDTC (107.5 months, 95% CI 36.6–NA), and PTC (94.0 months, 95% CI 94.0–NA). To examine the association between genetic alterations and OS, age (<65 and ≥65 years), sex, presence of lung or bone metastasis, pathological types, *BRAF* mutation, *RAS* mutations, *RET* or *NTRK* fusion genes, *TERT* promoter mutations, and *TP53* alterations were compared via log-rank test. In DTC and PDTC, cases with *BRAF* V600E mutation and *TP53* alterations had significantly shorter OS: the median OS was 107.5 months (95% CI NA–NA) in wild-type *BRAF*, 94.0 months (95% CI 70.2–NA) in *BRAF* V600E, 107.5 months (95% CI 94.0–NA) in wild-type *TP53*, and NA (95% CI 35.9–NA) in *TP53* alterations (Fig. 4B and 4C). Factors with log-rank test *P* < .2 in the Kaplan–Meier method were used to construct the Cox proportional hazards regression model. As shown in Table 5, *TP53* alterations remained an independent predictor of worse OS (hazard ratio = 2.205, 95% CI 1.135-4.283). Furthermore, we investigated whether the coexistence of *BRAF* and *TERT* promotor mutations affected OS in PTC; however, no significant difference was observed (*P* = .17, Fig. 4D). In ATC, there were no significant differences in any variables, and no genetic alterations associated with OS were observed.

**Table 5.** Cox proportional hazards regression modeling details in differentiated and poorly differentiated thyroid carcinoma

	Overall survival	*P*-value
	Hazard ratio (95% CI)	
Age (<65 vs ≥65)	1.543 (0.722-3.295)	.263
Bone metastasis (yes vs no)	0.626 (0.309-1.269)	.194
*BRAF* V600E mutation (yes vs no)	1.568 (0.761-3.230)	.223
*TERT* promoter mutation (yes vs no)	1.492 (0.734-3.034)	.269
*TP53* alterations (yes vs no)	2.205 (1.135-4.283)	.**020**

Abbreviation: CI, confidence interval.

*P* < .05 are shown in bold.

## Discussion

In this study, we used the C-CAT database, which is a national repository that contains information about only patients who underwent CGP testing (14). Because Japan has a public insurance system, CGP testing is performed for only in cases of advanced cancers that necessitate drug therapy. Therefore, DTC is classified as an advanced cancer with distant metastasis or an unresectable locally advanced cancer. Approximately 80% of patients with DTC and PDTC were treated with drugs including multikinase inhibitors. However, because ATC progresses rapidly and treatment options are limited, CGP testing is often performed at the time of diagnosis. Patients with ATC were therefore less likely than those with DTC to have distant metastases and a history of prior treatment at the time of registry.

The most common genetic abnormality in advanced thyroid cancer is *TERT* promoter mutations, which were observed in 66% of cases. According to a previous report, *TERT* promoter mutations were found in approximately 10% of patients with PTC, 17% of those with FTC, and 40% of those with ATC (20); these rates were lower than those in our study. *TERT* promoter mutations have a significantly high prevalence among cancers with aggressive histologic features, those in advanced stages, and those with distant metastases (20). The frequency of *TERT* promoter mutations may have been high in this study because we assessed only patients with advanced cancer. *TERT* promoter mutations were common in advanced cancers, both DTC and ATC, whereas *TP53* alterations were more abundant in ATC than in DTC. Oishi et al reported that *TP53* alterations are more frequently expressed in ATC components than in PTC components in cases of concurrent ATC and PTC (21). Furthermore, in a study of the gene profile of advanced cancers in the United States, *TERT* promoter mutations were common in DTC and ATC whereas *TP53* was abundant only in ATC (5); this gene profile was similar to that in this study. Comparing ATC and non-ATC, gene alterations such as tumor suppression genes or PI3K/AKT pathway genes were significantly more common in ATC, suggesting that gene mutations are accumulated more in ATC as previously reported (5). On the other hand, *RBM10*, which codes a RNA-binding protein that regulates the alternative splicing of pre-mRNA, was the only gene that was frequently mutated in DTC and PDTC. *RBM10* mutations have been reported to occur frequently in advanced DTC and PDTC, at a frequency of 7% to 11% (22), although they are infrequent in ATC (23). Furthermore, *RBM10* abnormalities are associated with RAI refractory and poorer survival in DTC (24). In this study, most cases with DTC were refractory to RAI, were treated with drug therapy, and were fatal. Therefore, *RBM10* mutations were more common in DTC and PDTC, similar to the previous reports.

Genetic abnormalities in thyroid carcinoma that appear early include many therapeutic targets. Of the patients with PTC, 84.7% had therapeutic targets, including *BRAF* V600E mutation (in 74.6%), *RET* fusion (in 6.5%), and *NTRK* fusion (in 2.9%). In the Cancer Genome Atlas study, *RET* and *NTRK* fusions were found in 6.8% and 1.2% cases, respectively, of mostly nonmetastatic thyroid cancers (25) and thus were as frequent as in our study. In another study, *RET* and *NTRK* fusions were found in 28% and 18%, respectively, of pediatric patients with PTC (26). The median age in this study was 71 years, which reflects the prevalence of cases among older adults who require drug therapy. Combined therapy with dabrafenib and trametinib has been reported to be effective in cases of ATC involving *BRAF* V600E mutation (8). *BRAF* V600E mutation was found in 47.7% of the patients with ATC in our study; this frequency is similar to that previously reported (5, 27). On the other hand, approximately half of the patients with ATC had no drug-therapeutic targets; thus, drugs must be developed for such cases. Another problem is that only 1.8% of patients with FTC with mainly *RAS* mutations have drug-therapeutic targets, and treatment options other than multikinase inhibitors are limited.

The most commonly used drug was lenvatinib, which was administered to 78% of patients who underwent drug therapy. In Japan, lenvatinib is also approved for ATC. Although lenvatinib was administered to 72% of the patients with ATC in this study who received drug therapy, their response rate was worse than that among patients with DTC. In the phase 3 SELECT study, lenvatinib significantly prolonged progression-free survival compared to placebo (7). The analysis of the SELECT study reported that cases with wild-type *BRAF* had a shorter progression-free survival than those with *BRAF* V600E mutation (28). However, in this study, TTF showed no difference regardless of the presence of *BRAF* or *RAS* mutations. This suggests that lenvatinib is effective regardless of the driver genes.

It has been reported that *BRAF* and *TERT* promoter mutations are associated with a high rate of malignancy in PTC (20). When OS from the start of drug therapy was evaluated, *BRAF* and *TP53* alterations were found to be factors for poor prognostics in DTC and PDTC. Furthermore, according to the Cox proportional hazards regression model, the presence of *TP53* alterations was an independent factor for poor prognosis. Although *TP53* alterations are known to be significantly abundant in cases of ATC (5), DTC with *TP53* alterations may reflect a state close to anaplastic transformation. Moreover, *TERT* promoter mutations have been shown to interact with *BRAF* mutations (29), and the coexistence of *BRAF* and *TERT* promoter mutations is a factor for poor prognosis in PTC (30, 31). This study is, to our knowledge, the first investigation of the influence of the coexistence of *BRAF* and *TERT* promoter mutations in patients with advanced thyroid carcinoma who require drug therapy, but we found no significant differences between patients with and without these coexisting mutations. The patients in this study had extremely advanced cancer, and *TERT* promoter mutations were observed in more than 60% of cases. The aggressiveness of these tumors may be associated with *TP53* alterations. In this study, the prognosis of patients with ATC did not differ between those who had and did not have *BRAF* V600E mutation, but the data were obtained in a situation in which access to *BRAF* inhibitors was limited. According to a single-center retrospective analysis, OS improved with the introduction of *BRAF*-directed therapy (27), and we hope that the approval of *BRAF* inhibitors will help improve prognosis.

One limitation of this observational study was that we used nonrandomized real-world data and did not intervene in cases of cancer at the time of database registration or treatment strategy. In the information registered by each medical institution, some data were missing, and it was not possible to match patient backgrounds and treatment strategies. Another limitation was selection bias, which occurred because we studied only patients who underwent CGP testing. However, in Japan, examination and treatment are restricted by the public insurance system; therefore, our real-world data included data from only patients with advanced cancers. Furthermore, all patients who underwent CGP tests within the public insurance system are registered in this database, which is quite large. Another feature of this database is that prognosis information is also registered.

In conclusion, we conducted a cohort study of advanced thyroid carcinoma in patients requiring drug therapy. The most common genetic abnormality was *TERT* promoter mutations, but we found no difference in prognosis with regard to the presence or absence of *TERT* promoter mutations or whether they coexisted with *BRAF* V600E mutation. The presence of *TP53* alterations was an independent factor for poor prognosis in advanced DTC and PDTC.

## Data Availability

Some or all datasets generated during and/or analyzed during the current study are not publicly available but are available from the corresponding author on reasonable request.

## Abbreviations

ATC: anaplastic thyroid carcinoma
CGP: comprehensive genetic profiling
CI: confidence interval
DCR: disease control rate
DTC: differentiated thyroid carcinoma
ECOG-PS: Eastern Cooperative Oncology Group performance status
F1CDx: FoundationOne® CDx
F1L: FoundationOne® Liquid CDx
FTC: follicular thyroid carcinoma
MSI: microsatellite instability
NA: not assessable
NCC: OncoGuide™ NCC Oncopanel System
ORR: overall response rate
OS: overall survival
PDTC: poorly differentiated thyroid carcinoma
PTC: papillary thyroid carcinoma
TMB: tumor mutation burden
TTF: time to treatment failure
